# Human papillomavirus infection among head and neck squamous cell carcinomas in southern China

**DOI:** 10.1371/journal.pone.0221045

**Published:** 2019-09-23

**Authors:** Guoying Ni, Kunsong Huang, Yi Luan, Zaizai Cao, Shu Chen, Bowei Ma, Jianwei Yuan, Xiaolian Wu, Guoqiang Chen, Tianfang Wang, Hejie Li, Shelley Walton, Fang Liu, Bobei Chen, Yuejian Wang, Xuan Pan, Xiaosong Liu, Ian H. Frazer

**Affiliations:** 1 The First Affiliated Hospital/Clinical Medical School, Guangdong Pharmaceutical University, Guangzhou, Guangdong, China; 2 Genecology Research Centre, University of the Sunshine Coast, Maroochydore DC, QLD, Australia; 3 Cancer Research Institute, Foshan First People’s Hospital, Foshan, Guangdong, China; 4 The Second Affiliated Hospital, Wenzhou Medical University, Wenzhou, Zhejiang, China; 5 Inflammation and Healing Research Cluster, School of Health and Sport Sciences, University of Sunshine Coast, Maroochydore DC, QLD, Australia; 6 The University of Queensland, Faculty of Medicine, Diamantina Institute, Translational Research Institute, Woolloongabba, QLD, Australia; Roswell Park Cancer Institute, UNITED STATES

## Abstract

Human papillomavirus (HPV) related tumours account for a significant proportion of head and neck squamous cell carcinomas (HNSCCs) in developed countries. They respond better to chemo- and radio-therapy, and have a better stage specific prognosis. To establish their prevalence in China, we assessed a series of histology confirmed HNSCCs collected in Zhejiang and Guangdong provinces by PCR for HPV DNA and by immunohistochemistry for p16 protein status. Among 303 HNSCCs, HPV DNA was detected in 26.4%, with HPV16 DNA in 71% of these. Of HNSCC located in the oropharynx, 38.55% (32/83) were HPV+ve. In this series, p16 status was a relatively poor predictor of HPV status as detected by PCR. The stage specific survival time of HPV+ HNSCCs was significantly longer than for HPV- HNSCC. HPV status should be assessed for oropharyngeal cancers in China to assist with appropriate management, and prophylaxis against HPV infection should be considered to reduce the incidence of this disease.

## Introduction

Head and neck squamous cell carcinoma (HNSCC) is the 6^th^ most common cancer worldwide [1, 2], with nearly 600,000 people diagnosed every year, and more than 300,000 deaths [3]. HNSCC is often associated with tobacco and alcohol use and with poor oral hygiene. HNSCCs are not uncommon in China: according to GLOBOCAN 2012, the estimated age standardized incidence rate in China is 2.7 per 100,000 [4], and a recent report, based on oropharyngal cancer (OPC) reported to 135 cancer registries during 2008–2012, estimated the age-standardized incidence of OPC as 2.22/100,000 person-years using the 2000 Chinese standard population (ASRIC and ASRMC) and 0.94/100,000 person-years using the 1985 Segi’s world standard population (ASRIW and ASRMW) [5].

Over the past decade, there has been a shift in the primary site distribution of HNSCC in western countries, with a steady increase in OPC and a decline in the cancers of the larynx and hypopharynx [1]. Persisting infection of the oropharynx and tonsil with HPV-16 is associated with a subset of OPC [6] that are of lower average age at onset, and are not strongly associated with alcohol and tobacco use. HPV associated OPC respond better to chemoradiotherapy than HPV negative OPC [6], and in the majority of studies, HPV associated OPC have a better survival compared with stage matched HPV negative OPC [7, 8].

Studies of large cohorts of patients to investigate incidence and risk factors for HPV related HNSCC across Asia are limited. In a retrospective study of HNSCC in Taiwan, the overall prevalence of HPV infections was 19% [9]. In a study conducted in Hong Kong examining patients with HNSCC over a 5-year period from 2005 to 2009, 20.8% (43/207) of OPC and 29.0% (36/124) of tonsillar SCC were associated with HPV16 [10]. However, in a study in south China, high-risk HPV infection was found in only 7.5% (17/228) of HNSCC [11].

China is experiencing rapidly social and economic change, and this change may ultimately influence the incidence of cancer, especially cancers associated with life style and sexual behaviours. It is therefore useful to monitor the risk factors for HNSCC in China, including tumour HPV status, to better prevent and treat HNSCCs. Here, we report on a retrospective study across three hospitals of Guangdong and Zhejiang provinces, in the developed southern region of China, designed to establish the prevalence of HPV+ and HPV- HNSCCs with special attention to oropharyngeal cancers. We show that HPV infection contributes significantly to the development of HNSCC in this region.

## Materials and methods

### Ethics statement

The human ethics for the current project were approved by Foshan First People’s Hospital, The First Affiliated Hospital of Guangdong Pharmaceutical University and the 2^nd^ Affiliated Hospital of Wenzhou Medical University. The ethical approval codes from Foshan, Guangzhou and Wenzhou hospitals were L2016 (13), GYFY201703, and LCKY2018-59 respectively. After the ethics was approved by the Human Ethics Committee of the Foshan First People’s Hospital on 15 December 2016, the project firstly started from Foshan on 22 December 2016. As we wished to include more samples, colleagues of Wenzhou and Guangzhou joined our team and obtained the ethics approvals respectively. The ethnics of Guangzhou was approved by the Human Ethics Committee of the First Affiliated Hospital of Guangdong Pharmaceutical University on 17 January 2017, and patient samples’ collection started from 6 February 2017. The ethics of Wenzhou was approved by the Human Ethics Committee of the Second Affiliated Hospital of Wenzhou Medical University on 14 December 2018, patient samples’ collection started from 15 December 2018.

### Samples and patient cohort information

Patients with biopsy confirmed HNSCC, presenting to Foshan First People’s Hospital, to the First Affiliated Hospital of Guangdong Pharmaceutical University, Guangdong Province or to the Second Affiliated Hospital of Wenzhou Medical University, Zhejiang province between 2005 to 2018 (Guangzhou), 2009 to 2017 (Wenzhou), 2012 to 2017 (Foshan) were included in this retrospective study. The study was started from 2016. Authors had no access to information that could identify individual participants during or after data collection. Criteria for the inclusion of subjects were:

(1) Presentation with a new primary squamous cell carcinoma of larynx, hypopharynx, tonsil, gingiva, palate, tongue, buccal, epiglottis, mouth floor, or base of tongue;
(2) No other primary cancers present;

Exclusion criteria were

(1) Metastatic tumour, tumours from elsewhere that presented as a metastasis in the oropharynx, were excluded;
(2) Patients with incomplete medical records, or inadequate tissue sample for DNA extraction for PCR or for immunohistochemical analysis;
(3) Concurrent nasopharyngeal cancer related to Epstein–Barr virus (EBV);
(4) Human immunodeficiency virus (HIV) infection.

Of 388 patients meeting the entry criteria, 85 were excluded, mostly because there were inadequate tissue samples and incomplete patient information (**Table 1**).

**Table 1:** Patients’ HNSCC information from three hospitals.

Parameters	Values (Foshan%)	Values (Guangzhou%)	Values (Wenzhou%)	Values (Total%)
**Age at diagnosis in years (SD)**	59 (9.8)	53 (13.9)	62 (11.62)	58 (12.68)
**Sex(n[%])**				
Male	84(88.0)	92(69.70)	71(93.42)	247(81.52)
Female	11(12.0)	40(30.30)	5(6.58)	56(18.48)
**Smoking use(n[%])**				
Never smoker	32(33.7)	81(61.36)	18(23.68)	131(43.23)
Former smoker	10(10.5)	0	0	10(3.30)
Current smoker	51(53.7)	50(37.88)	57(75)	158(52.15)
Not recorded	2(2.1)	1(0.76)	1(1.32)	4(1.32)
**Mean smoking pack-years (SD)**	31(11.5)	Not recorded	Not recorded	Not recorded
**Alcohol use(n[%])**				
No	53(55.8)	103(82.58)	28(36.84)	184(60.72)
Yes	40(42.1)	29(17.42)	47(61.84)	116(38.28)
Not recorded	2(2.1)	0	1(1.32)	3(1.0)
**Initial tumor stage (n[%])**				
**Tumor category**				
Tis	1(1.1)	0	1(1.32)	2(0.66)
T1	11(11.6)	23(17.43)	22(28.95)	56(18.48)
T2	36(37.9)	32(24.24)	21(27.63)	89(29.37)
T3	16(16.8)	47(35.60)	19(25)	82(27.06)
T4	24(25.3)	30(22.73)	11(14.47)	65(21.45)
Tx	7(7.4)	0	2(2.63)	9(2.97)
**Nodal category(n[%])**				
N0	28(29.5)	81(61.36)	46(60.52)	155(51.16)
N1	22(23.2)	31(23.48)	3(3.95)	56(18.48)
N2	30(31.6)	16(12.12)	22(28.95)	68(22.44)
N3	3(3.2)	4(3.03)	3(3.95)	10(3.30)
Nx	12(12.6)	0	2(2.63)	14(4.62)
**Metastasis category (n[%])**				
M0	80(84.2)	130(98.48)	75(98.68)	285(94.06)
M1	2(2.1)	2(1.52)	0	4(1.32)
Mx	13(13.7)	0	1(1.32)	14(4.62)
**Primary treatment(n[%])**				
Surgery	75(78.9)	132(100.0)	76(100.0)	283(93.4)
Chemoradiation	12(12.6)	0(0.0)	0	12(3.96)
Not recorded	8(8.4)	0	0	8(2.64)

Remark: AJCC Cancer Staging, 7th edition.

### HPV DNA extraction and PCR analysis

Five to seven slices (5 μm) from each sample were collected, and DNA extraction was performed using TaKaRa MiniBEST FFPE DNA Extraction Kit (TaKaRa Bio Group, Japan) [12, 13], using a deparaffinization method without xylene. Paraffin was eliminated during a single step of incubation in mineral oil at 80°C for 1 min. Then, 20 μl of Proteinase K (20 mg/ml) and 10 μl of RNase A (10 mg/ml) were added and the sample held at 56°C for one hour. Samples were eluted into 30 μl of elution buffer and stored at -20°C.

PCR was performed using Promega (GoTaq Green Master Mix) PCR kit, with 12.5 μl of PCR mixed buffer, 2 μl of primer, 2 μl of DNA template and 25 μl Nuclease-Free Water. Forty cycles of amplification were performed on a Bio LifeEco PCR machine after an initial step of 3 minutes denaturation at 94°C. For HPV16 E7, the primers were forward (5′-CCCAGCTGTAATCATGCATGGAGA-3′), reverse (5′-GTGTGCCCATTAACAGGT CTTCCA-3′). For Non-HPV16, the primers were MY09 (5′-CGTCCMARRGGAWACTGATC-3′), MY11 (5′-GCMCAGGGWCATAAYAATGG-3′). Where M = 50%A or 50%C; W = 50%A or 50%T; Y = 50%C or 50%T; R = 50%A or G. Therefore, the primer MY09 and MY11 are a mixture of 4x3x2x1 sequences(MY09), 3x2x1(MY11) sequences [14]. For β-globin, the primers were forward (5′-AGGAGAAGTCTGCCGTTACTG‐3′), reverse (5′‐CCGAGCACTTTCTTGCCATGA-3′). In each batch of tests, Nuclease-Free water was used as a negative control and HPV16+ cervical cancer samples were used as a positive control. Conditions for HPV16 and β-globin PCR were 94°C for 2 minutes, followed by denaturation at 94°C for 30 seconds, annealing at 58°C for 45 seconds, and extension at 72°C for 1 minute for a total of 35 cycles. For non-HPV16, PCR conditions were 94°C for 2 minutes, followed by denaturation at 94°C for 30 seconds, annealing at 55°C for 1 minute, and extension at 72°C for 1 minute for a total of 35 cycles. PCR amplicons were analyzed by 2% agarose gel containing ethidium bromide and identified under UV light.

The concentration of the extracted DNA were measured by NanoDrop (Thermo Scientific NanoDrop 2000/2000c). The concentration of 90% of DNA samples exceeded 30 ng/μl. All DNA samples had OD values between 1.8 and 2.0 at 260 nm, and all samples used for the study tested positive for β-globin.

### HPV type determination

A PCR-RDB HPV genotyping assay (Yaneng Biotech, Guangzhou, China) that can identify 17 High Risk-HPV types (16, 18, 31, 33, 35, 39, 45, 51, 52, 53, 56, 58, 59, 66, 68, 73 and 82) and 6 LR-HPV types (6, 11, 42, 43, 81 and 83) was used. Briefly, the L1 consensus HPV PGMY09 (5′-CGTCCMARRGGAWACTGATC-3′) and PGMY11 (5′-GCMCAGGGWCATAAYAATGG-3′) primers were used to amplify 5 μl of extracted DNA in a 20 μl reaction volume. HPV was amplified in an ABI Veriti96 PCR machine under the following conditions: 50°C for 15 minutes, 95°C for 10 minutes, followed by denaturation at 94°C for 10 seconds, annealing at 45°C for 90 seconds, and extension at 72°C for 30 seconds for a total of 40 cycles [15, 16]. After amplification, HPV genotyping was done by reverse-dot-blot (RDB) hybridization on the strips fixed with 23 different HPV type specific probes, followed by color development with tetramethylbenzidine (TMB). All steps were performed manually in the presence of appropriate controls provided by the manufacturer. The blue spots on the strip were judged positive/negative by direct observation.

### Immunohistochemistry

The p16 protein is a cyclin-dependent kinase (CDK) inhibitor that decelerates the cell cycle by inactivating the CDKs that phosphorylate retinoblastoma (Rb) protein. p16 monoclonal antibody (E6H4), immunohistochemistry kits and DAB chromogenic reagents were purchased from Roche Biotechnology, USA. Paraffin sections were deparaffinized and subjected to immunohistochemically staining using a fully automated immunohistochemical stainer (Benchmark XT from Roche, USA).

The results of p16 immunohistochemistry were evaluated and confirmed by two pathologists. A tumour was regarded as p16 positive if the nuclei and/or the cytoplasm of squamous epithelial cells were stained. According to the proportion of positive cells, p16 was divided into 4 levels: cells without staining or less than 5%: negative (-); stained cells between 5% and 25% is considered (+), stained cells between 25% and 50% is (++), stained cells between 50% and 75% is (+++), and stained cells larger than 75% is (++++) [17].

### Statistical analysis

The survival rates of various HNSCCs was compared by Log-Rank analysis. Risk factors between HPV+ and HPV- oropharyngeal cancers and concordance of HPV and p16 staining were analysed by Fisher’s exact test using a Prism 8 software (Graphpad).

## Results

### Demographic and clinical characteristics of the studied patients

This study comprises a retrospective analysis of 388 patients presenting to 3 major Chinese hospitals over a period of up to 10 years with a diagnosis of primary squamous head and neck region cancer. Of these, 303 had sufficient clinical data and pathological material available to allow inclusion in the analysis. The clinical and demographic findings of the studied patients are summarized in **Table 1**, and showed the expected predominance of males, and of tobacco users. Cancers of the larynx/ hypopharynx (32%) and tongue (37%) accounted for most of the included subjects, although the spectrum of cancer sites was significantly different between the three participating hospitals (**Table 2**). Patients were divided into oropharyngeal cancers and other HNSCC groups. Cancers from base of tongue (33, 10.89%), tonsil (29,9.57%), soft palate (18, 5.94%) and epiglottis (3, 0.99%) were considered as oropharyngeal cancers, while cancers from Larynx or Hypopharynx (97, 32.01%), TIPS/Corpus/Linguae (96, 31.68%), Gingiva (12, 3.96%), mouth floor (6, 1.98%), buccal (5, 1.65%), hard palate (4, 1.32%) were grouped as other HNSCCs.

**Table 2:** Tumour HPV and p16 status.

Tumour location	Tumour status		TOTAL
	HPV+ {(p16+)%/(p16-)%)}	HPV- {(p16 +)%/(p16-)%}	
Oropharyngeal	32(28.12%/71.88%)	51(11.76%/88.24%)	83
Not oropharyngeal	48(37.5%/62.5%)	172(17.44%/82.56%)	220
All tumour totals	80(33.75%/66.25%)	223(16.14%/83.86%)	303

### HPV prevalence

**Fig 1:**
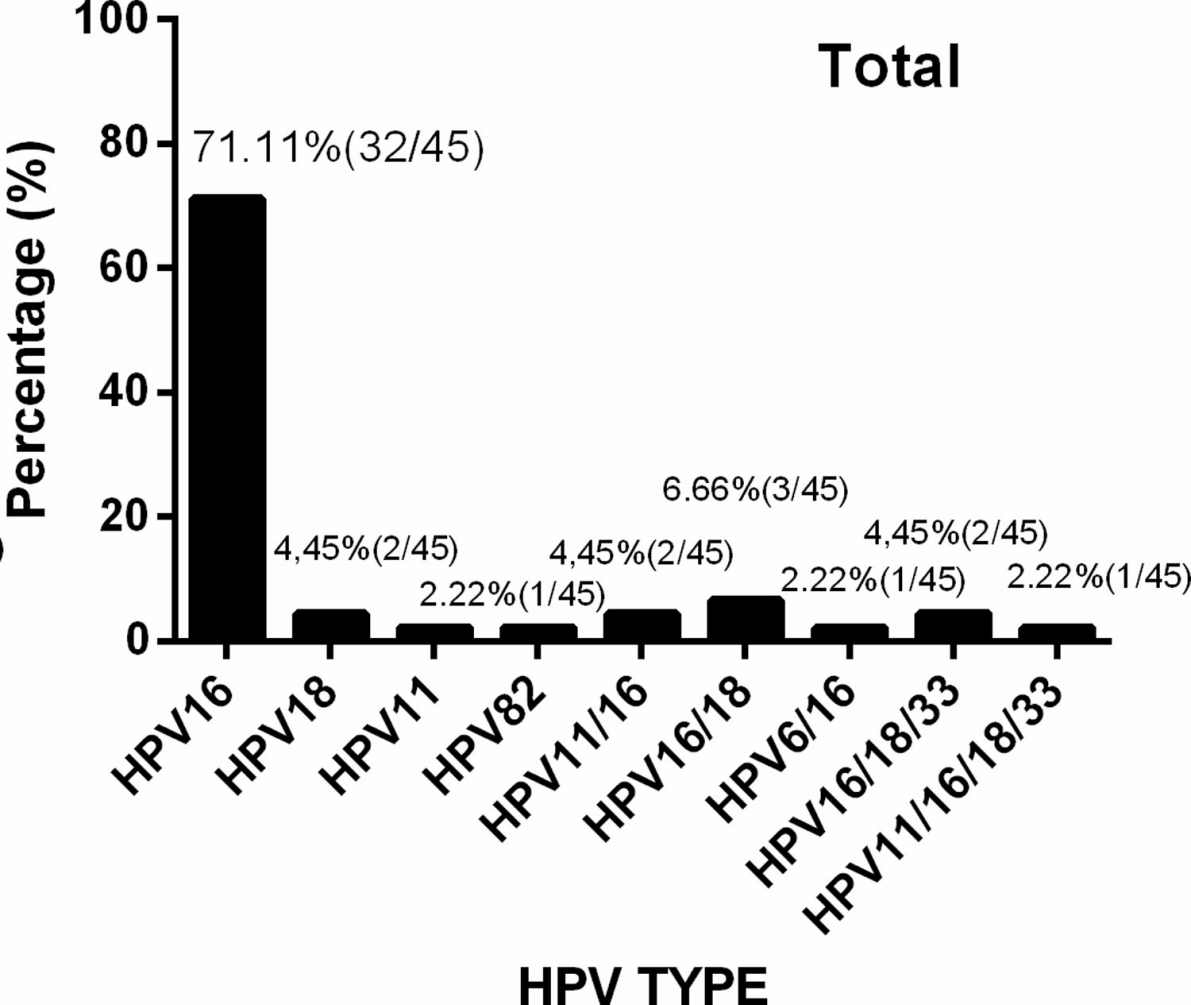
HPV subtypes in HPV related HNSCCs. Tumour samples from each contributing hospital that tested positive for HPV16 E7 with HPV16 specific primers were further tested for type specific α-HPV DNA by HPV L1 gene specific PCR and reverse blotting of product against a range of probes specific for α-papillomaviruses. Samples from (a) Wenzhou (b) Foshan.

**Fig 2:**
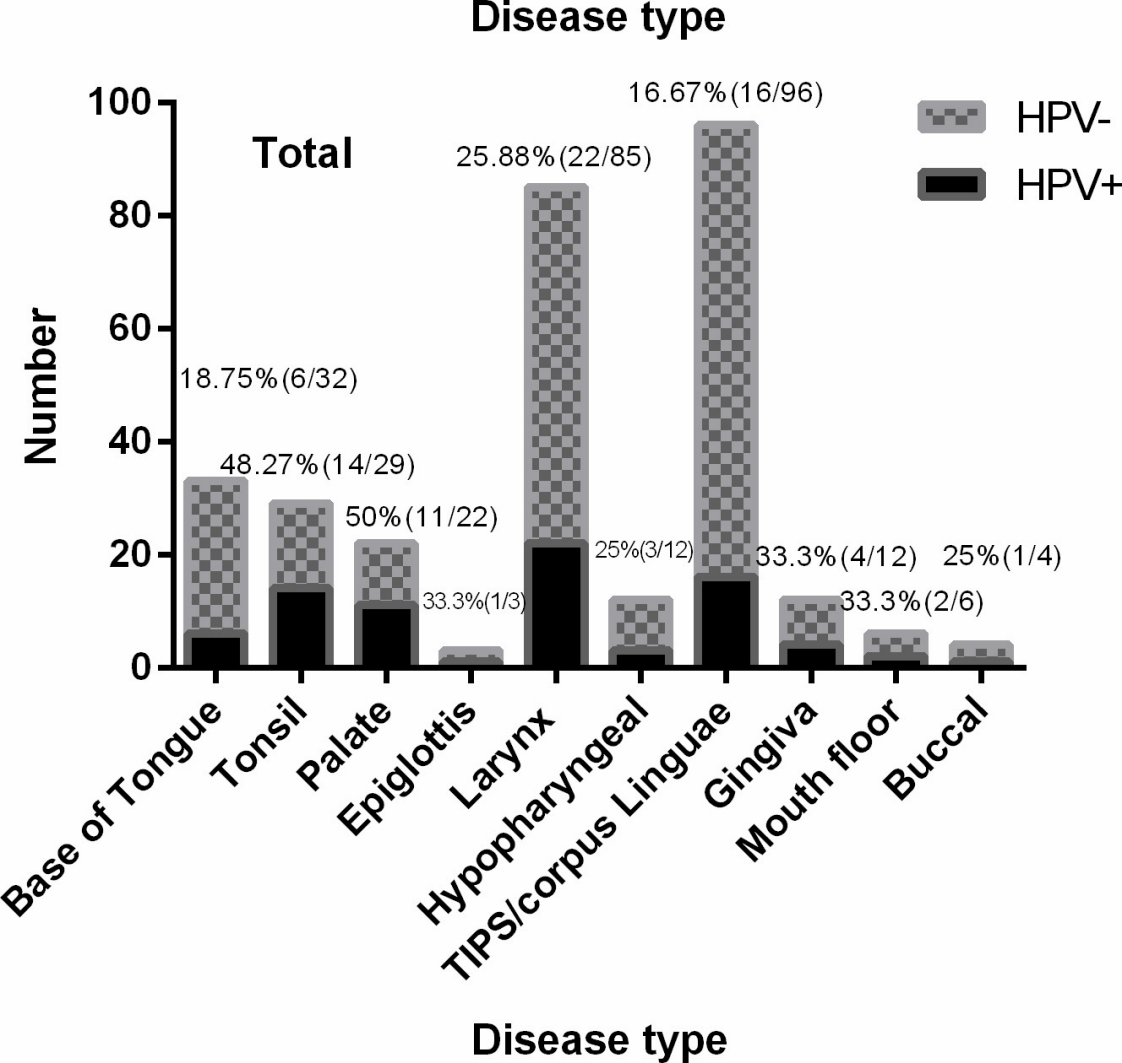
Detection of HPV in HNSCC according to tumour site. For each HNSCC site, the total number of cancers at that site, and the fraction that were HPV+ve, is shown for (a) Guangzhou (b) Foshan (c) Wenzhou and (d) aggregated data.

To determine the role of HPV infection in the genesis of the studied cancers, paraffin embedded tissue was tested for HPV DNA. HPV was first tested using a genotype specific PCR test, and then further tested by PCR reverse dot blot hybridization for HPV subtypes. HPV 16 was the dominant HPV subtype observed in patients from Guangzhou and from Foshan, accounting for 91.3% and 88.8% of total HPV+ samples respectively, with overall HPV16+ rate of 91.1% amongst HPV positive tumours. Only 3 subtypes, 16, 18, 82, were detected in samples from Guangzhou, while 5 subtypes, 16, 18, 11, 6, 33, including low risk subtypes, were detected from Foshan samples. Multiple HPV subtypes were detected in tumours a small fraction of patients (**Fig 1**), and HPV was detected in some cancer specimens from all anatomical regions considered in the current study (**Fig 2**).

### Concordance of HPV and p16 staining

There was the expected wide range of p16 staining across the sampled tumours, with 36 subject biopsies showing greater than ≥75% of tumour cell staining, 23 showing 50%-75% and 25 showing some positive cells but with <50% staining. As p16 staining is widely used as a surrogate marker for HPV infection in HNSCC, and HPV infection is most commonly associated amongst HNSCC with oropharyngeal cancer we analysed those tumours located as arising the from tonsil, palate, epiglottis and lingual root, considered as oropharyngeal, and compared them with the other HNSCC cancers, considered as non-oropharyngeal cancers (**Table 2**). Some concordance of p16 staining with HPV detection was observed across all samples (Fisher’s exact test; p = 0.0013), and within the non-oropharyngeal cancers (p = 0.005), but concordance for p16 and HPV was non-significant within the tumours classed as oropharyngeal (p = 0.08). Concordance of HPV detection with p16 staining was similarly observed when analysis was conducted separately for each hospital source.

### Risk factor analysis and survival time between HPV+ and HPV- oropharyngeal cancers

**Fig 3:**
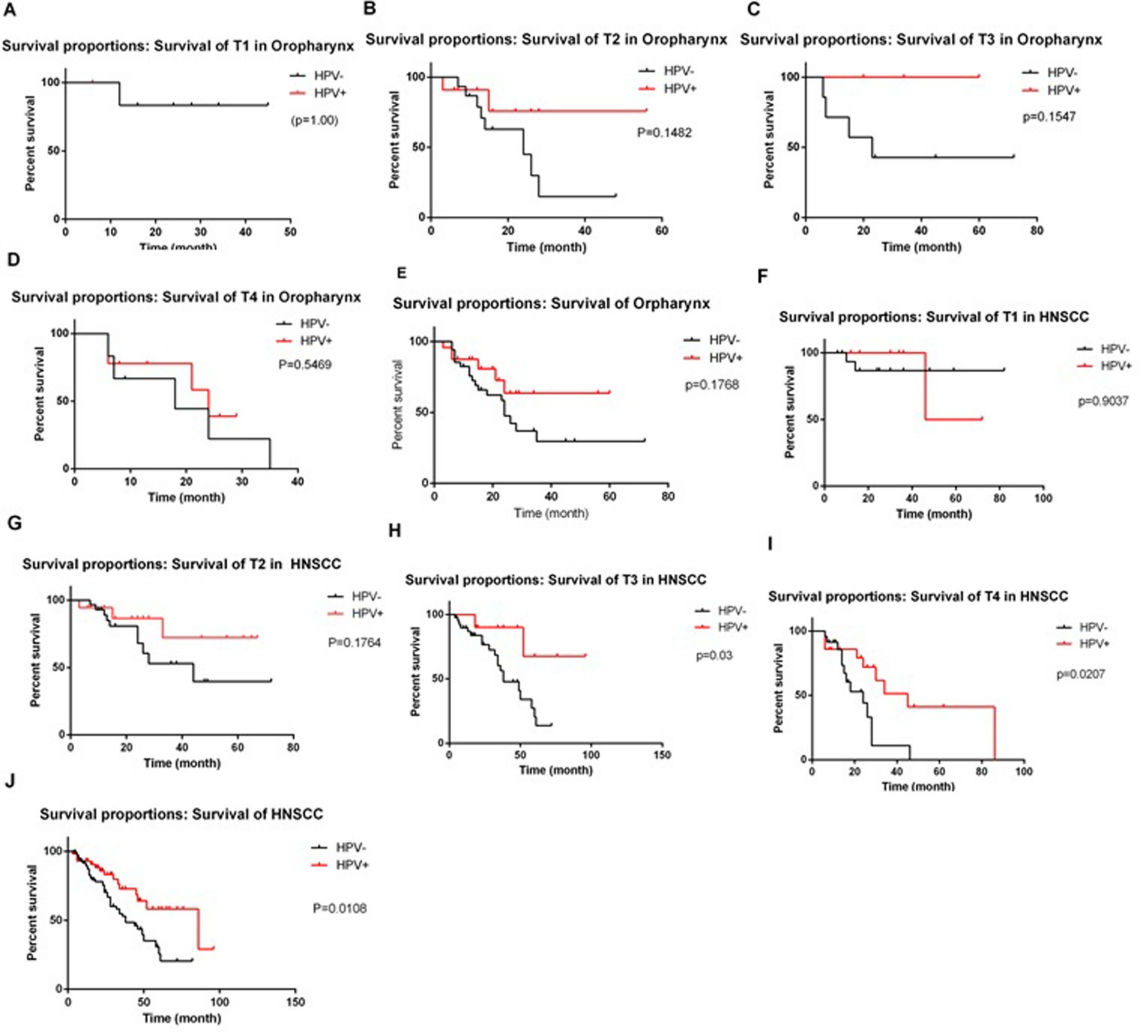
HPV status as a predictor of survival following treatment. Oropharyngeal cancer survival is shown by tumour stage at treatment (a) T1 (b) T2 (c) T3 (d) T4 (e) all tumours. HNSCC survival is shown by tumour stage at treatment (f) T1 (g) T2 (h) T3 (i) T4 (j) all tumours. The significances of the survival differences by log-rank analysis are shown.

No significant association of HPV positivity amongst the entire studied tumour population was observed with age (p = 0.3; fisher’s exact test) sex (p = 0.7), smoking habit (p = 0.6), or alcohol consumption (p>0.9) (**Table 3**). The difference in mean survival time between HPV+ and HPV- HNSCCs was analyzed for 165 patients with survival data, of whom 83 were oropharyngeal cancer patients (**Fig 3**). HPV status, age, gender and location of the tumour were not different between the 165 analysed patients compared with the 305 patients included in the current study. The mean survival of HPV+ve HNSCC cancer patients was significantly longer than for HPV-ve HNSCCs patients (p = 0.001). If patients survival was analysed by their initial tumour stages of T1-T4, the mean survival time for HPV+ HNSCC patients was significantly longer than HPV- HNSCCs for patients presenting with T3 (p = 0.03) and T4 (p = 0.007) stage tumours, but not for patients presenting with T1 and T2 stage tumours. If only oropharengneal cancer patients are compared, there are no statistical differences between HPV+ve and HPV-ve OPCs, whether they are divided into T1-T4 stages, or calculated together (**Fig 3**).

**Table 3:** Factors predictive of HPV positive tumours.

Factor	HPV-positive	HPV-negative	Total	P values (Fisher’s exact test)
**Drinking**				**1.00**
Alcohol	**12**	**23**	**35**	
Non-Alcohol	**20**	**28**	**48**	
**Sex**				**0.75**
Male	**29**	**42**	**71**	
Female	**3**	**9**	**12**	
**Tobacco**				**0.64**
Smoking	**18**	**33**	**51**	
Non-smoking	**14**	**18**	**32**	
**Age (Year)**				**0.31**
35–65	**26**	**34**	**60**	
≥ 65	**6**	**17**	**23**	

## Discussion

In this study, we examined factors that might predispose to HNSCC amongst patients presenting with primary HNSCC in major teaching hospitals in the more developed southern regions of China over the last 15 years. The majority of patients presenting with HNSCC were male, and more than 50% were current smokers, in keeping with the known aetiology of HNSCC in studies elsewhere. HNSCC are closed related with smoking and alcohol consumption, most patients in the western world present at over 60 years of age. HPV+ OPC patients in our current study account for 43.75% of non-smoker, 41.66% of non-alcohol drinker and 43.33% of those age between 35–65, higher than smoker (35.29%), alcohol drinker (24.28%) and those age over 65 (26.08%). Although not statistically significant, the results are in agree with published literature that HPV+HNSCC are more commonly observed in younger age, non-smoking and non-alcohol drinking, and sexual active patients [18]. No obvious reason for the relative predominance of OPC amongst HNSCC in Foshan, not seen in the other two centres, could be determined from the available historic data.

HPV prevalence in HNSCCs and HPV related survival of HNSCCs have been published around the world and in China. The prevalence of HPV+ HNSCCs among HNSCCs varies greatly, from 0 to 70% [19–22]. HPV+HNSCC Factors contributing HPV+HNSCCs are not fully understand, while immune suppression and concurrent HIV infection may contribute to the prevalence of HPV+HNSCCs [20–22]. The study of large cohort of HNSCCs in developed area of southern China is uncommon. In a meta-analysis of HPV related HNSCC in China, HPV16 related HNSCCs were reported, the overall pooled HPV-16 prevalence among head and neck cancer cases was 24.7% (20.2–29.3%); most studies were conducted in eastern China (numbers of study = 16, 57.14%), and the remaining studies as 6 (21.43%) studies in central China, 5 (17.86%) studies in western China and 1 (3.57%) study in northeastern China. In southern China, only laryngeal cancers were studied in Guangdong province, while no HPV related HNSCCs in Zhejiang province was conducted [21].

Here, we show that HNSCC in the three surveyed hospitals in mainland China were commonly associated with evidence of HPV infection, and that this association was not limited to OPC, as is more commonly seen in Europe and the USA [23]. We also show that amongst the tested Han Chinese population, p16 was not a particularly useful maker of the HPV status of HNSCC, in contrast to most studies amongst Caucasian populations. In some studies, HPV related HNSCCs account for over 70% of total HNSCCs [24, 25]. The overall percentage of HPV+ve HNSCC in the current study was 26.4%, without major differences between the three study centres. HPV16 infection is most strongly associated with base of tongue and tonsillar SCCs, and patients from our study contains a larger group of HNSCCs of other anatomical regions, which may explain the current prevalence of HPV16 infection observed our patient samples. The HPV+HNSCC is 26.4% by PCR, 20.7% by p16 staining, 38.3% if p16+ and HPV PCR+ are counted; further study should focus on which technique is more accurately reflect the HPV infection status in Chinese Han patients given the concordance between p16 and HPV PCR is low. It was found in a meta-analysis that PCR is more sensitive in detecting HPV DNA than *In situ* hybridisation (ISH) if paraffin embedded samples were used [20]. Prospective study using fresh samples may better reflect the status of the HPV infection.

Previous studies of HNSCC amongst Chinese in Taiwan reported an overall prevalence of HPV infections of 19%, with a trend toward decreasing rates from 2004 to 2011 [9]. In a Hong Kong study, using an E6/7 mRNA marker of oncogenic involvement, 20.8% (43/207) of OPSCC and 29.0% (36/124) of tonsillar SCC was associated with HPV [10]. However, in a study in southern mainland China study, high-risk HPV infection was found in only 7.5% (17/228) of HNSCCs, and only a small proportion of samples had evidence of viral integration (5.3%, 12/228) or E6/7 mRNA expression (4.4%, 10/228) [11]. In a study in Northern China, 211 laryngeal squamous cell tumours were analysed by PCR, in situ hybridization and p16 immunohistochemistry, and 132 (62.6%) were positive for HPV DNA (HPV+) [26]. In another study from Northern China, formalin-fixed and paraffin-embedded tissues of 93 head and neck SCC patients were included. Presence of HPV16/18 oncoprotein in tumour tissues was assessed by immunohistochemistry (IHC) with HPV16/18 E6-specific antibodies. Amongst 93 patients, the total positive rate of HPV genome and its encoding products in the tested samples was 44.1% [27]. The investigation of HPV infection among HNSCCs in Chinese Han patients from above and our study can be improved by including large cohort of patients from different anatomical regions of HNSCC, and by conducting prospective studies. We are recruiting more patients with the base of tongue and tonsil, to address specifically the HPV infection and compare with other regions of HNSCCs.

HPV infection related HNSCC are aetiologically different from HPV non-related HNSCCs, especially OPC which respond better to chemo- and radiotherapy and usually survive longer [7, 24, 28]. In developed countries, the incidence of HPV -ve HNSCCs is falling in association with lesser use of tobacco products and reduced alcohol consumption [24, 25]. However, in the current study, an inverse association of the HPV status of tumours and use of alcohol or tobacco was not observed. HPV-16 is the dominant HPV subtype related to HNSCCs in most published research [11, 24]. Various studies have reported that oral and tonsillar epithelial cells can be immortalized by full-length HPV 16 or its E6/E7 oncogenes; transgenic mouse models have revealed that HPV16 E6/E7 strongly increases susceptibility to oral and oropharyngeal carcinomas [24]. As expected, HPV 16 was also the dominant HPV subtype observed both in Guangzhou and Foshan; samples from Guangzhou and Foshan had HPV16 rates of 91.3% and 88.8% of total HPV+ve samples respectively, with overall HPV16+ rate 91.1%. Only 3 subtypes, 16, 18, 82, were detected in samples from Guangzhou, while subtypes, including low risk subtypes, 11, 6, were detected from Foshan samples. This discrepancy probably reflects socio-economic differences between the catchment areas of the two hospitals. In a recent study, HPV type 35 was found as the 2^nd^ most dominant HPV type in OPC tumours in USA [29], which is different from ours, probably geographic, ethnicity differences that contribute to the discrepancy.

Only 40–50% of patients with HNSCC will survive for 5 years from diagnosis, despite optimal surgical, chemo- or radio-therapy treatment [28]. However, HPV+ve cancers have a better prognosis, particularly if presenting without metastasis[30], such that T4 N0 HPV+ve OPC are recognized to have similar prognosis to T1 N0 HPV-ve tumours [31]. In the current study, the overall survival time of HPV+ OPC was significantly longer than those of HPV-oropharyngeal cancers in patients presenting with T3/ T4 tumours, confirming that HPV related HNSCC patients respond better to chemo- and radio-therapy compared with HPV negative tumours in China as elsewhere.

HPV related HNSCCs may be prevented by the HPV prophylactic vaccine. HPV16 E7 specific immunotherapy may also benefit HPV+ HNSCC patients, if efficacy of a HPV16 E7 specific immunotherapy is high enough [32–34]. The data from the current study suggest that as HPV related HNSCCs account for significant proportions of total HNSCCs in southern China, HPV status should be tested for routinely for HNSCC and preventive and therapeutic procedures against HPV should be considered.

## Supporting information

S1 File Attachment+3+Points+extracted+from+images+for+analysis(1). (XLSX)
